# Supplementary material for: Inflammatory factors and risk of lung adenocarcinoma: a Mendelian randomization study mediated by blood metabolites
Source: Front Endocrinol (Lausanne). 2024 Aug 27;15:1446863. doi: 10.3389/fendo.2024.1446863 (PMC11384989; doi:10.3389/fendo.2024.1446863)
Supplement: Supplementary file 1 [file DataSheet1.zip › supplementary files/S2/GCST90200875/sensitivity-analysis.pdf]

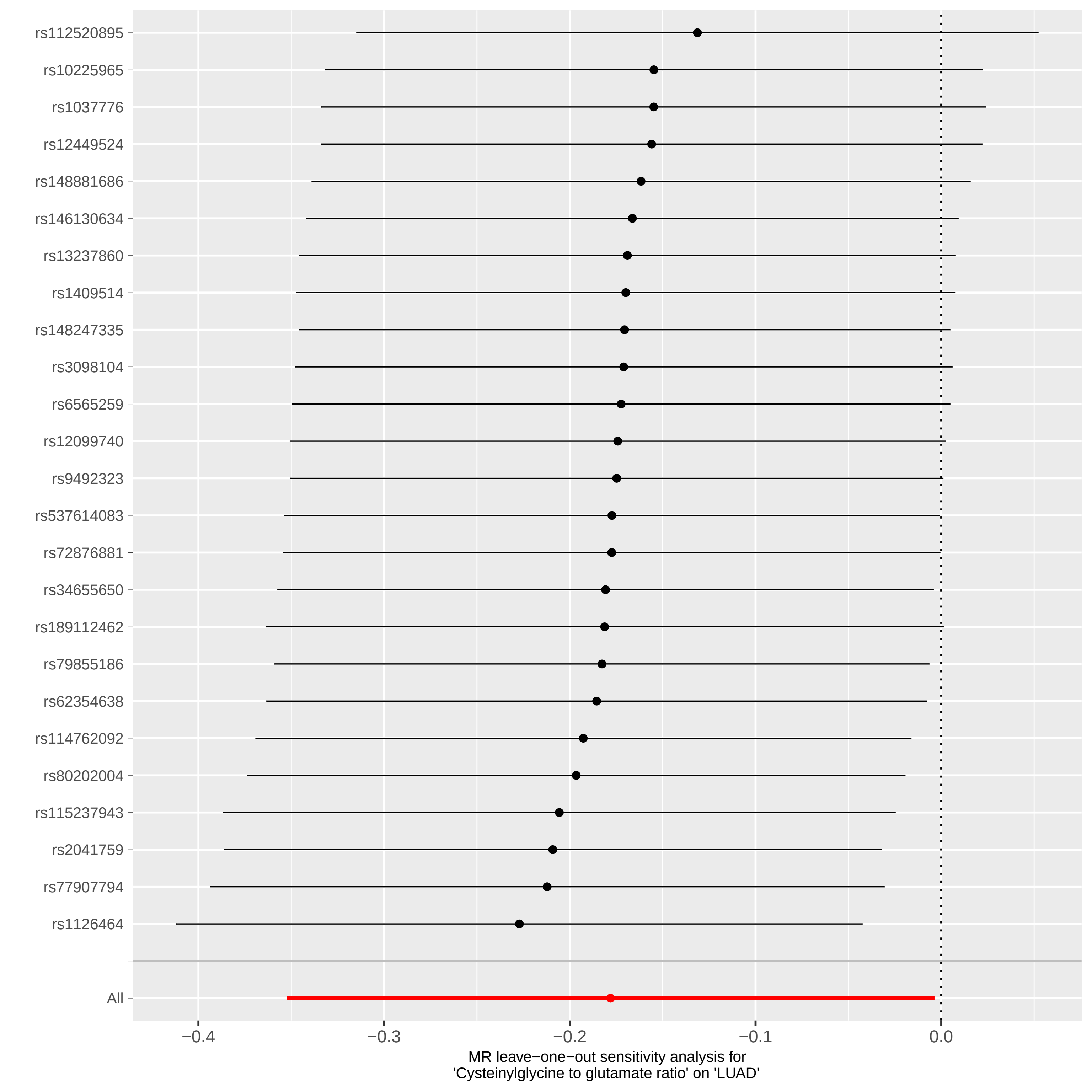
rs112520895
rs10225965
rs1037776
rs12449524
rs148881686
rs146130634
rs13237860
rs1409514
rs148247335
rs3098104
rs6565259
rs12099740
rs9492323
rs537614083
rs72876881
rs34655650
rs189112462
rs79855186
rs62354638
rs114762092
rs80202004
rs115237943
rs2041759
rs77907794
rs1126464
All
−0.4
−0.3
−0.2
−0.1
0.0
MR leave−one−out sensitivity analysis for
'Cysteinylglycine to glutamate ratio' on 'LUAD'
